# A Possible Mechanism of the Primary Open Angle Glaucoma

**Published:** 2013

**Authors:** Bijay Kumar Parida

**Affiliations:** Faifa General Hospital, Gizan, Kingdom of Saudi Arabia

**Dear Editor,**

Universally, it is an estimation that 60 million individuals with glaucomatous optic neuropathy. In addition, there are an estimated 8.4 million glaucomatous patients who are blind. These patients are expected to increase to 80 million and 11.2 million by the year 2020. Statistically, glaucoma has been considered as the second leading cause of blindness in the world [1].

The universal definition of glaucoma is that it is an optic neuropathy with assorted etiology, which disturbs the optic nerve head, thereby resulting in visual loss and permanent impairment of visual function [2]. On the other hand, primary open angle glaucoma is an optic neuropathy that is commonly associated with increased intraocular pressure. High intraocular pressure leads to retinal ganglion cell death and characteristic cupping of the optic disc [3].

The etiology, pathogenesis and mechanism of optic nerve damage in primary open angle glaucoma have not been clearly investigated. We propose that:

1. As intraocular pressure rises, it causes perforation in anterior hyaloid face.

2. Vitreous imparts maximum pressure on optic nerve head, followed by macula. Therefore, Bjerrum scotoma is seen early in primary open angle glaucoma.

3. With the rise of intraocular pressure, a knuckle of vitreous fills the optic cup. When the intraocular pressure is reduced, this knuckle of vitreous does not reverse itself. As the vitreous imbibes liquid, capillaries are compressed and optic nerve fibers are afflicted with ischemic changes.

Based on our hypothesis, complete vitrectomy inducing posterior vitreous detachment and releasing the impacted vitreous from the cup of optic nerve is perceived as a viable treatment in the cases who are otherwise not responding to medical intervention. 

With rise of intraocular pressure, micropores are produced in anterior hyaloid face, through which aqueous humor enters into vitreous and imparts pressure on optic nerve head and macula. This results in damage of optic nerve fibers and ganglion cells of macula simultaneously. The vitreous is pushed into the optic cup causing a rise of intraocular pressure and stays within optic cup permanently. 

Furthermore, the eye becomes hydrated due to damage of anterior hyaloid face even though the intraocular pressure is reduced to 10-12 mm Hg. Pressure is exerted on capillaries and occludes them partially, damaging optic nerve fibers.

Our hypothesis can be directly tested in live animal models to shed further light on the subject.
